# Dermatofibrosarcoma presenting as a nodule in the breast of a 75-year-old woman: a case report

**DOI:** 10.1186/1752-1947-5-503

**Published:** 2011-10-05

**Authors:** Olivier Cottier, Maryse Fiche, Jean-Yves Meuwly, Jean-François Delaloye¹

**Affiliations:** 1 Département de Gynécologie-Obstétrique et Génétique, Centre Hospitalier Universitaire Vaudois, Lausanne, Suisse; 2 Institut Universitaire de Pathologie, Centre Hospitalier Universitaire Vaudois, Lausanne, Suisse; 3 Service de Radiodiagnostic et de Radiologie Interventionnelle, Centre Hospitalier Universitaire Vaudois, Lausanne, Suisse

## Abstract

**Introduction:**

Dermatofibrosarcoma protuberans is a rare neoplasm of soft tissues and its location in the breast is extremely uncommon. Confusion is possible with other primary breast lesions.

**Case presentation:**

A 75-year-old Caucasian woman presented with a mass in her left breast 21 years after being diagnosed with invasive ductal carcinoma of the right breast, treated by a right mastectomy and axillary dissection followed by radiotherapy and breast reconstruction. Mammography revealed a dish-shaped skin nodule formation in the upper outer quadrant of her left breast. Echography confirmed the presence of a lesion measuring 1.4 × 0.8 cm. Based on imaging, the diagnosis was a probable angiosarcoma. Due to the presence of a pacemaker for cardiac arrhythmia and full anticoagulation therapy for a pulmonary embolism, magnetic resonance imaging and a biopsy were not done. We proceeded directly to a quadrantectomy and the final diagnosis revealed a dermatofibrosarcoma protuberans, 1. 8 cm in its greatest microscopic dimension, located 0.1 cm from the upper surgical margin. To ensure the wide resection margins required for this type of neoplasm, a re-excision was performed.

**Conclusion:**

A dermatofibrosarcoma protuberans of the breast is an uncommon discovery. The aim of this case report is to highlight the importance of the surgical procedure in cases of the discovery of dermatofibrosarcoma protuberans. Re-excision may be necessary to ensure adequate resection margins.

## Introduction

Dermatofibrosarcoma protuberans (DFSP) is a rare neoplasm of soft tissues described in 1924 by Darier and Ferrand as "progressive recurrent dermatofibroma" and by Hoffmann in 1925 as 'dermatofibrosarcoma protuberans'. This tumor is a dermal spindle cell tumor of intermediate malignancy characterized by a slow evolution, a significant risk of local recurrence and a low rate of metastasization [1]. DFSP typically presents during early or middle adult life in all parts of the body, although more frequently on the trunk, extremities, and head and neck [1]. Its location in the breast is extremely rare and very few cases have been reported in the literature. Confusion is possible with other primary breast lesions [2,3].

## Case presentation

**Figure 1:**
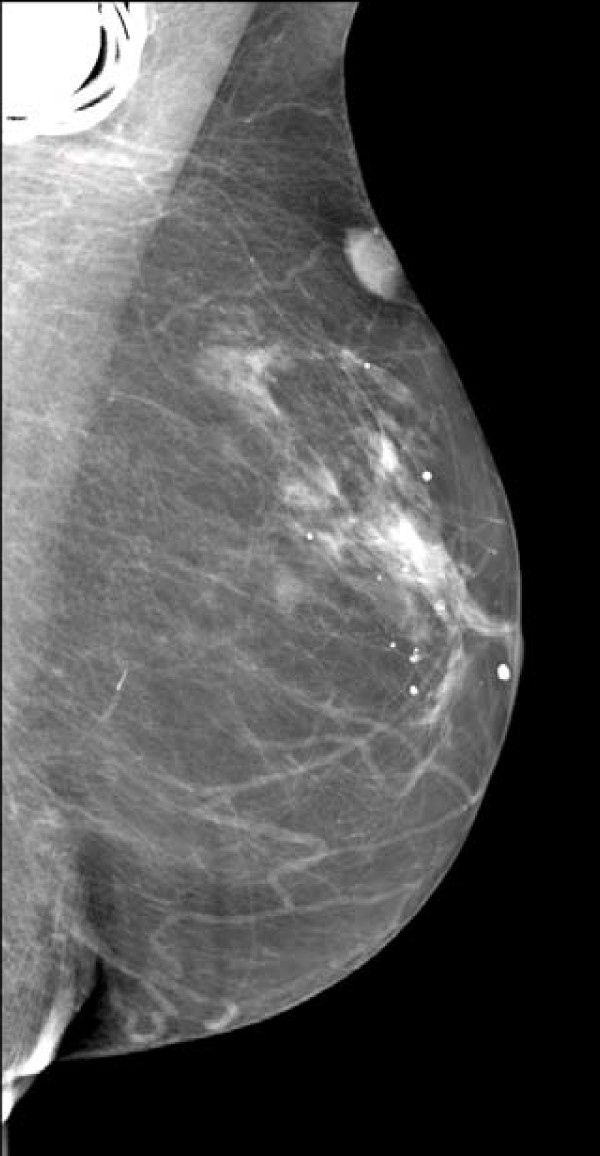
**Mammography**. Mediolateral oblique view; appearance of a nodular formation of her left breast.

**Figure 2:**
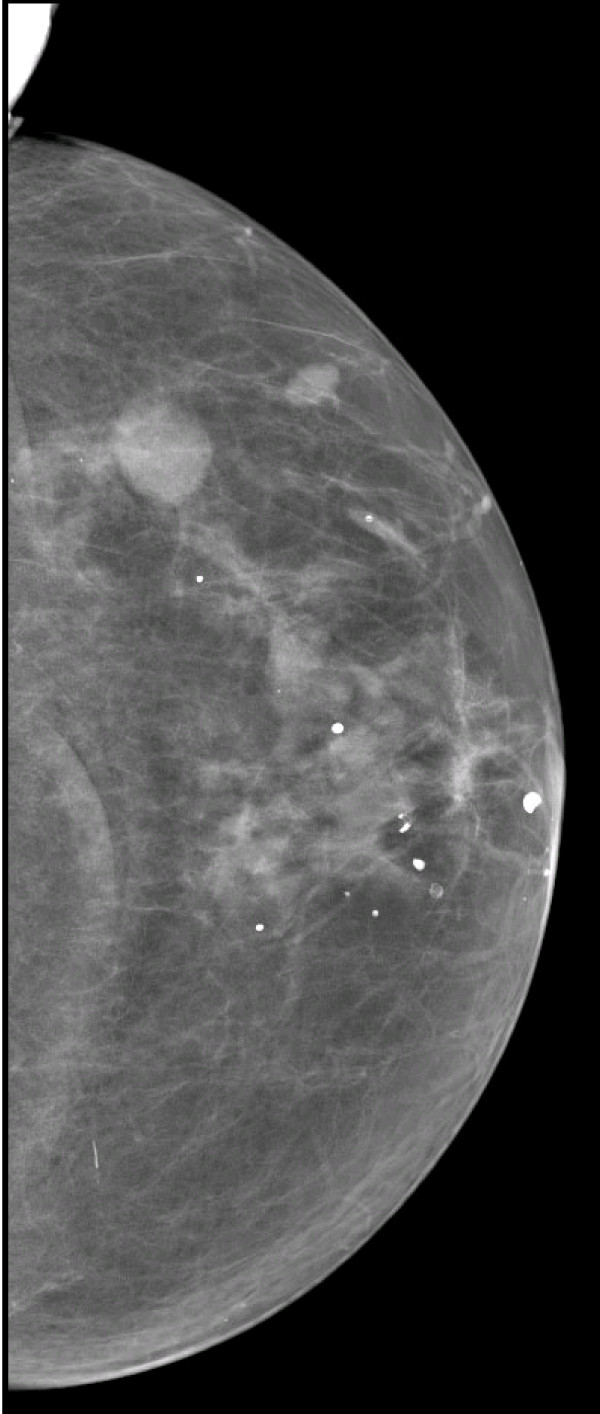
**Mammography**. Craniocaudal view.

**Figure 3:**
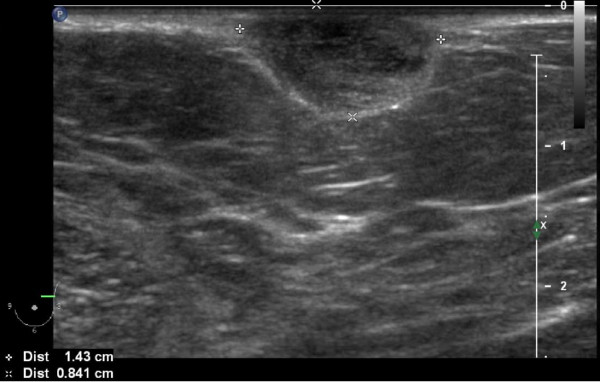
**Ultrasonography**. Nodular lesion in her left breast measuring 1.4 × 0.8 cm.

**Figure 4:**
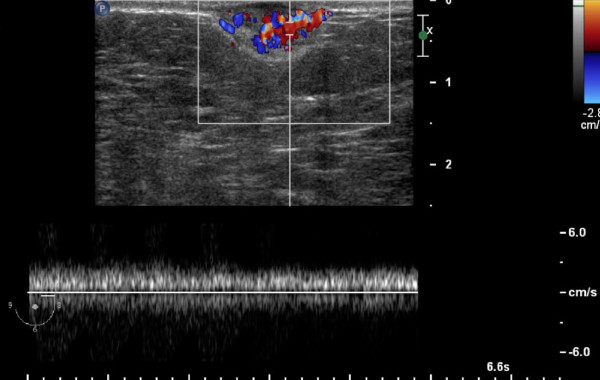
**Ultrasonography**. Highly vascular lesion in the Doppler mode.

We present here the case of a 75-year-old Caucasian woman, who 21 years ago underwent a right mastectomy and axillary dissection followed by radiotherapy and breast reconstruction with a prosthesis for invasive ductal carcinoma of her right breast, and now presented with a mass in her left breast. Mammography showed a dish-shaped skin nodule in the upper outer quadrant of her left breast (Figures 1 and 2). Echography confirmed the presence of a lesion measuring 14 × 8 mm. Based on imaging, the diagnosis was a probable angiosarcoma (Figures 3 and 4). She has a history of hypertension, a pacemaker for cardiac arrhythmia and was also treated with acenocoumarol for a pulmonary embolism two years ago. Magnetic resonance imaging (MRI) was not feasible due to the pacemaker. We proceeded to a quadrantectomy after modifying anticoagulation therapy. Her postoperative recovery was uneventful.

**Figure 5:**
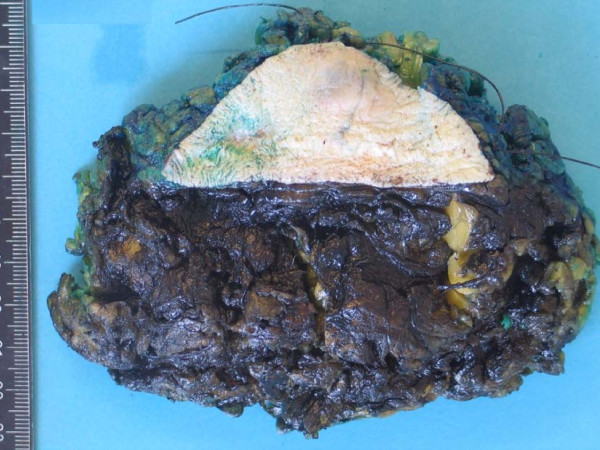
**Pathology (gross)**. The quadrantectomy specimen (11 × 11 × 4 cm).

**Figure 6:**
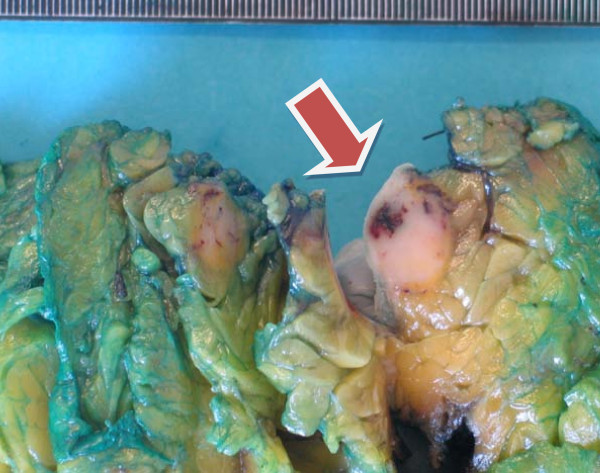
**Pathology (gross)**. Well-defined bluish nodule of 1 × 1 cm, with areas of hemorrhage (arrow).

**Figure 7:**
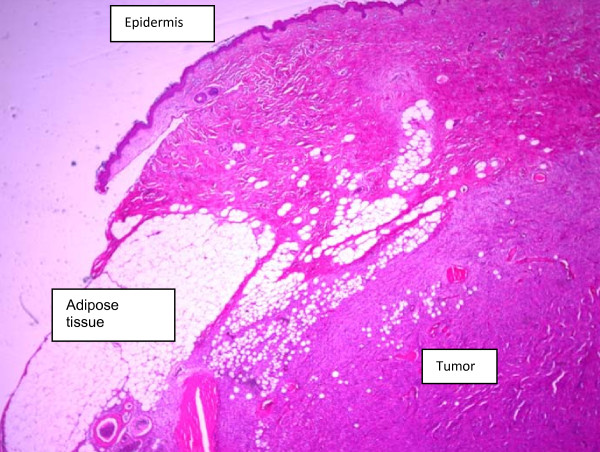
**Pathology (microscopy)**. The tumor infiltrates the hypodermal adipose tissue.

**Figure 8:**
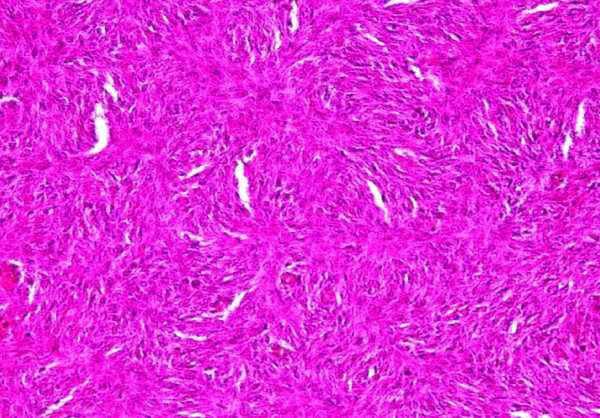
**Pathology (microscopy)**. Proliferation of spindle cells with elongated nuclei and moderate nuclear pleomorphism; fewer than four mitoses per 10 high power fields have been counted.

**Figure 9:**
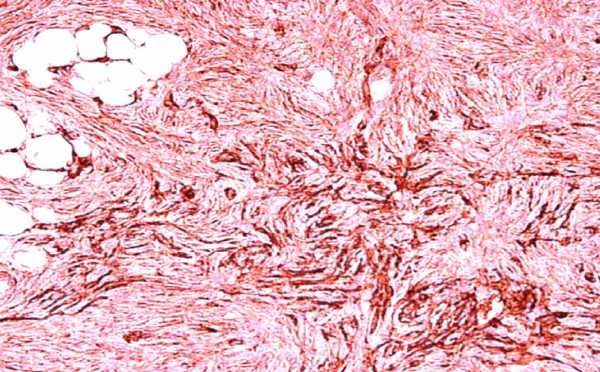
**Pathology (immunohistochemistry)**. Tumor cells diffusely and strongly expressed the CD34 antigen.

At gross examination, the specimen measured 11 × 11 × 4 cm and harbored a 1 × 1 cm well delineated dermal nodule close to the upper surgical margin. The cut section showed a solid whitish tumor with foci of hemorrhage (Figures 5 and 6). Microscopic examination revealed a proliferation of bland spindle cells arranged in a storiform pattern extending into hypodermal fat (Figures 7 and 8). These cells diffusely and strongly expressed the CD34 antigen, and were negative for CD31 and S-100 protein (Figure 9). The diagnosis was DFSP; 1.8 cm in its greatest microscopic dimension located 0.1 cm from the upper surgical margin. To ensure the wide resection margins required for this type of neoplasm, a re-excision was performed, up to the pectoral muscle fascia and including some muscle fibers. Pathology examination showed no residual tumor. This re-excision allowed for additional safety margins of at least 5 cm. No additional treatment was done. Our patient is well with no evidence of recurrence one year after surgery.

## Discussion

DFSP represents about 1% of soft tissue sarcomas with an estimated incidence of 0.8 to 5.0 cases per million per year [2,4]. Forty-seven percent of DFSP cases occur on the trunk [1]. Breast localization of DFSP is rare [3,5]. In most cases, mammography reveals a dense lesion without fat or calcification. Ultrasound exploration identifies the lesion in the dermis or subcutaneous tissue and the use of Doppler shows hypervascularization of the area [3]. Even in a patient receiving anticoagulation therapy, core biopsy is an option: this biopsy is essential to obtain a diagnosis in order to plan a one-time wide excision. MRI may be helpful to define the depth of infiltration of the tumor [5].

Pathologic examination reveals monotonous spindle cells arranged in a storiform pattern, extending to the hypodermal fat in a typical honeycomb pattern [6]. The differential diagnosis includes mainly benign fibrous histiocytoma, and also neurofibroma and myxoid liposarcoma [1]. DFSP cells are typically diffusely positive for CD34, which indicates a close link between this neoplasm and normal CD34 positive dermic dendritic cells [1]. Genetic abnormalities associated with DFSP include a supernumerary ring chromosome, corresponding to the low amplification of sequences of chromosomes 17 and 22, and/or the presence of t(17;22), a balanced reciprocal translocation. This translocation fuses the platelet-derived growth factor beta-chain (PDGF-beta) gene to the collagen type 1, alpha 1 gene [6]. The fusion protein, which has a PDFG-beta-type effect, participates in cell proliferation and can be blocked by tyrosine kinase inhibitors[7].

Safety margins should be of several centimeters of healthy tissue and should have an anatomical border not invaded at depth. The appropriate distance between free surgical margins and the tumor, however, is not established. Some authors recommend Mohs surgery; micrographic surgery using the microscope to trace out the ramifications as describe by Mohs in 1978. This offers a complete evaluation of the peripheral and deep margins using frozen section or accelerated standard histology [8,9]. Wide first intention local excision may be preferable in the parts of the body where it is easy (like trunk and limb), resulting in an overall shorter procedure [9]. A plastic surgeon should be present if wound closure difficulties are anticipated.

Local recurrence rate varies between 1.6% and 50% depending on the type of surgery used [6,10,11]. Mohs surgery results in extremely low local recurrence rates and, accordingly, a cure rate of up to 98.5% [12]. Regional and distant recurrences are infrequent (regional lymph node metastases and distant metastases, principally in the lung), estimated at less than 5% of cases [11]. Complementary radiation therapy or chemotherapy seem not to bring any benefit [13]. However, specific tyrosine kinase inhibitors (for example imatinib, which inhibits the PDGF-beta receptor) appear promising [7]. Long-term follow-up requires strict monitoring every six to twelve months with ultrasound and biopsy in cases of suspected recurrence. The five-year survival rate of patients with DFSP is over 99% [14,15].

## Conclusion

Breast localization of DFSP is extremely uncommon and can mimic a primary breast tumor. As in other locations of DFSP, surgical excision with adequate resection margins is recommended to ensure local control of the disease. A plastic surgeon should be present if difficulty with the wound closure by first intention is to be expected.

## Consent

Written informed consent was obtained from the patient for publication of this case report and any accompanying images. A copy of the written consent is available for review by the Editor-in-Chief of this journal.

## Competing interests

The authors declare that they have no competing interests.

## Authors' contributions

OC and JFD analyzed and interpreted the patient data. MF performed the histological examination. JYM performed the imaging and ultrasonography. OC was a major contributor in writing the manuscript. MF wrote the pathology section. All authors read and approved the final manuscript.
